# Proteomic signatures of protected *APOE* ε4 carriers reveal causal pathways associated with delayed Alzheimer's disease onset

**DOI:** 10.1002/alz.71688

**Published:** 2026-08-13

**Authors:** Yann Le Guen, Junyoung Park, Andrés Peña‐Tauber, Michael D. Greicius

**Affiliations:** ^1^ Department of Medicine Quantitative Sciences Unit Division of Computational Medicine Stanford University Stanford California USA; ^2^ Department of Neurology and Neurological Sciences Stanford University Stanford California USA

**Keywords:** Alzheimer's disease, *APOE* ε4, biomarker discovery, delayed disease onset, Global Neurodegeneration Proteomics Consortium, loss‐of‐function burden, Mendelian randomization, plasma proteomics, resilience, target prioritization

## Abstract

**INTRODUCTION:**

*APOE* ε4 is the strongest common genetic risk factor for Alzheimer's disease (AD), yet many carriers remain cognitively unimpaired into late life. We tested whether protected ε4‐first plasma proteomics could identify proteins associated with delayed clinical onset.

**METHODS:**

We analyzed harmonized Global Neurodegeneration Proteomics Consortium (GNPC) plasma proteomics. Protected ε4 carriers (ε3/ε4 ≥75 years; ε4/ε4 ≥65 years; Clinical Dementia Rating [CDR] score = 0; *n* = 456) were compared with ε4 carriers with AD (*n* = 1096). Protein‐wise models adjusted for age, sex, ε4 dosage, and plasma proteomic principal components. Top signals were integrated with loss‐of‐function burden testing and plasma/cerebrospinal fluid Mendelian randomization.

**RESULTS:**

Protected ε4 status was associated with 721 protein measures. Integrated analyses prioritized LILRA5, DBI, BPNT1, PTEN, EPHA1, and PCDH10 as ε4‐modified candidates and OMG, SELENOW, VAT1, and TPPP3 as broader AD‐related signals. TREM2 and ACE were also identified.

**DISCUSSION:**

This strategy highlights immune, synaptic, metabolic‐stress, and myelin/axonal pathways that may delay AD onset.

## Highlights

- Some *APOE* ε4 carriers remain cognitively unimpaired into late life.
- A protected ε4‐first proteomic design identified 721 associated proteins.
- Human genetics prioritized candidate modifiers including LILRA5 and EPHA1.
- Findings implicate immune, synaptic, metabolic, and axonal mechanisms.
- Studying protected high‐risk individuals may reveal prevention‐relevant targets.

## 1 BACKGROUND

Alzheimer's disease (AD) is the most common cause of dementia and a major global public health challenge. Recent estimates indicate that tens of millions of people worldwide are living with dementia, and the number of individuals across the AD continuum is expected to rise sharply with population aging.^1^, ^2^ Contemporary biological frameworks define AD by its underlying pathologic processes rather than by symptoms alone, emphasizing that amyloid beta (Aβ) deposition, tau pathology, and neurodegeneration accumulate over a prolonged preclinical period before overt dementia emerges.^3^, ^4^ This extended interval between biological onset and clinical diagnosis creates an important opportunity for prevention‐oriented discovery, particularly if mechanisms that delay symptom onset can be identified in living humans.

Genetic risk for AD is substantial, but not deterministic. The *APOE* ε4 allele remains the strongest common genetic risk factor for late‐onset AD, with dose‐dependent effects on risk and age at onset.^5^, ^6^ However, *APOE* ε4 is neither necessary nor sufficient for AD: Many ε4 carriers never develop dementia, and estimated absolute risks vary by age, sex, and ancestry.^5^ Studies of *APOE* ε4 carriers therefore provide a particularly informative setting in which to search for protective factors that modify the relationship between inherited risk and clinical outcome.^7^, ^8^, ^9^

Conceptually, this strategy draws on the literature distinguishing resistance from resilience in AD. Resistance refers to lower‐than‐expected pathology given risk, whereas resilience refers to better‐than‐expected cognitive or clinical function despite pathology.^7^ In a research setting where biomarkers are unavailable and subjects cannot be classified as either resistant or resilient, we favor the more agnostic term “protected.” In many observational cohorts, however, complete amyloid and tau biomarker data are unavailable, making delayed clinical onset among genetically high‐risk individuals a pragmatic phenotype for enrichment of protective biology. In the *APOE* ε4 context, defining cognitively unimpaired older ε4 carriers as protected therefore provides an operational framework to identify biological factors associated with delayed dementia onset.^7^, ^8^

Blood‐based proteomics offers a scalable strategy for discovering such biology in living humans. Large affinity‐based platforms, including aptamer‐based assays such as SomaScan, can quantify thousands of plasma proteins simultaneously and have shown suitable reproducibility for population‐scale biomarker discovery studies.^10^, ^11^ These technologies enable systems‐level analyses in cohorts that are substantially larger than those typically available for cerebrospinal fluid (CSF) or neuroimaging studies.^10^, ^12^, ^13^ The Global Neurodegeneration Proteomics Consortium (GNPC) recently harmonized multi‐cohort plasma proteomic data at unprecedented scale, improving power for biomarker and target discovery across neurodegenerative diseases and aging.^12^ However, an important limitation of conventional case–control proteomic analyses is that many proteins associated with AD diagnosis or cognitive decline may reflect downstream responses to neurodegeneration, inflammation, frailty, medication, or comorbidity rather than upstream modifiers of disease onset.^7^, ^12^, ^13^

**RESEARCH IN CONTEXT**

1. **Systematic review**: Previous studies framed AD protection in terms of resistance, resilience, and reserve and examined why some *APOE* ε4 carriers remained cognitively intact despite elevated genetic risk. Large‐scale plasma proteomics has also emerged as a promising approach for AD biomarker and target discovery. However, these approaches have not been widely integrated in a design centered specifically on clinically protected older ε4 carriers, with additional prioritization through human genetics.
2. **Interpretation**: In this study, a protected ε4‐first plasma proteomic framework identified proteins associated with preserved clinical status among older *APOE* ε4 carriers. Integration with broader disease‐related proteomic analyses, loss‐of‐function burden testing, and MR prioritized proteins involved in immune signaling, metabolic regulation, axonal biology, and synaptic maintenance. These findings suggest that studying clinically protected high‐risk individuals may help distinguish proteins associated with delayed onset from those that primarily reflect downstream disease processes.
3. **Future directions**: Replication in independent ε4‐enriched cohorts and longitudinal studies will be important to confirm whether these proteins are associated with delayed conversion rather than survivor bias. Integration with amyloid and tau biomarkers may help distinguish resistance‐related from resilience‐related mechanisms. Experimental follow‐up will also be needed to determine whether the prioritized proteins represent causal mediators, compensatory responses, or biomarkers of protective pathways.

Human genetics can help address this limitation through causal triangulation. Large protein quantitative trait loci (pQTLs) resources have now mapped genetic regulators of plasma and CSF protein levels, providing instruments for causal inference and target prioritization.^14^, ^15^, ^16^ Two‐sample Mendelian randomization (MR) using pQTLs can strengthen inference regarding whether altered protein abundance is more likely to lie upstream of disease risk, particularly when combined with complementary evidence from co‐localization and disease genetics.^15^, ^16^, ^17^ In parallel, rare‐variant gene‐based analyses in large sequencing cohorts can identify genes in which predicted loss‐of‐function variants are enriched in AD, offering an independent line of evidence for biological relevance.^18^, ^19^ Integrating proteomic associations with orthogonal human genetic evidence therefore increases confidence that prioritized proteins are not merely correlates of disease state but candidate drivers or modifiers of disease onset.^15^, ^16^, ^18^, ^19^

Here, we applied a protected APOE ε4‐focused framework to large‐scale GNPC plasma proteomics to identify proteins associated with preserved clinical status among older ε4 carriers. We first compared cognitively unimpaired older ε3/ε4 and ε4/ε4 carriers with ε4 carriers affected by AD, then contextualized these signals in population‐wide AD and APOE ε4‐by‐AD proteomic models. Finally, we integrated the proteomic findings with high‐confidence loss‐of‐function burden testing and plasma/CSF MR to prioritize proteins supported by complementary human genetic evidence. We hypothesized that proteins supported across these layers would nominate biological processes associated with delayed clinical onset in APOE ε4 carriers.

## 2 METHODS

### 2.1 Study design overview

**FIGURE 1:**
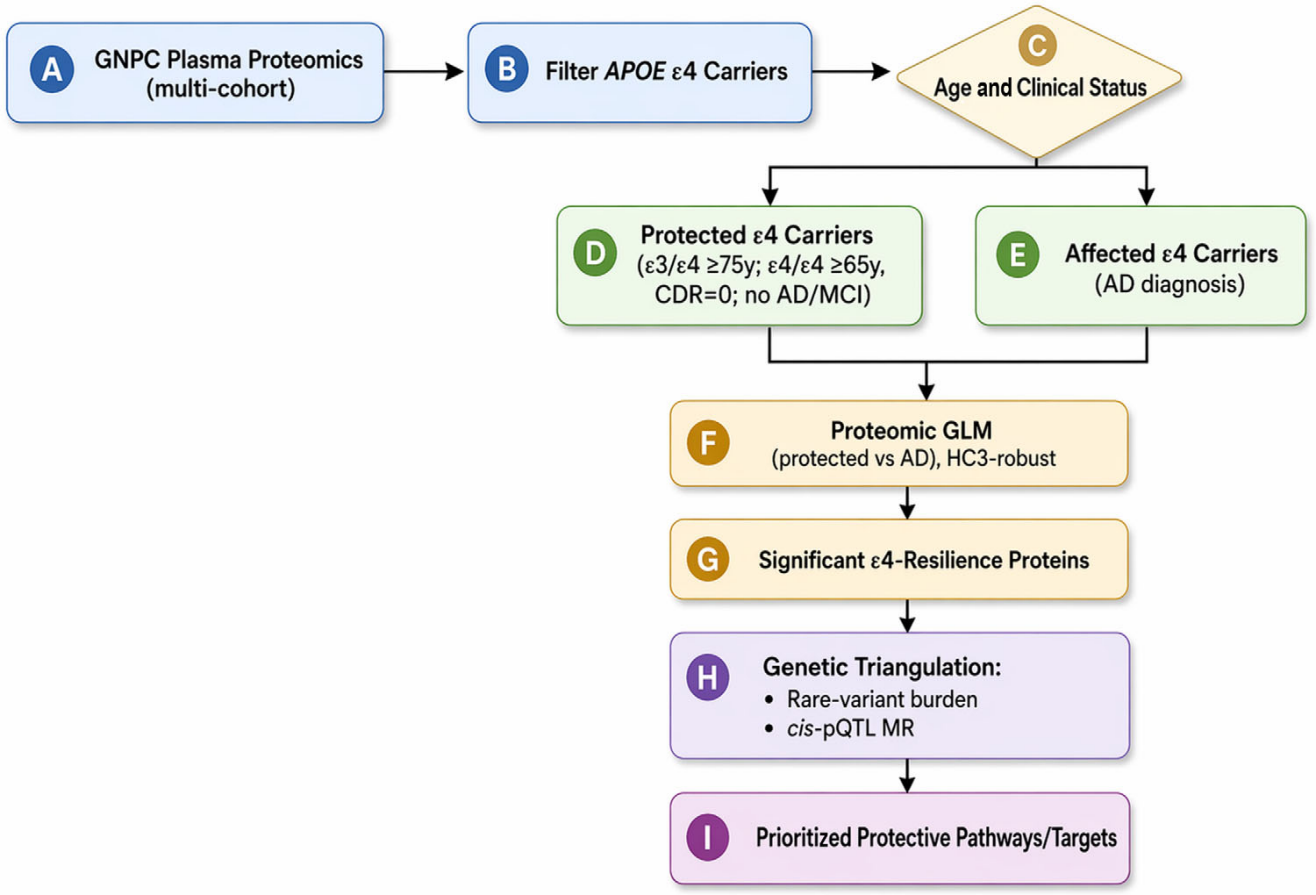
Protected ε4‐first plasma proteomics and genetic triangulation framework. Harmonized GNPC EDTA plasma proteomic data were used to compare clinically protected older APOE ε4 carriers with APOE ε4 carriers with Alzheimer's disease. Protein‐wise linear models identified protected ε4‐associated proteins, which were then interpreted in the context of population‐wide diagnosis and APOE ε4‐by‐diagnosis analyses and prioritized through orthogonal human genetic evidence, including high‐confidence loss‐of‐function burden testing and plasma/cerebrospinal fluid Mendelian randomization. GNPC, Global Neurodegeneration Proteomics Consortium.

We applied a protected ε4‐first plasma proteomic framework in the Global Neurodegeneration Proteomics Consortium (GNPC) to identify proteins associated with delayed clinical onset among *APOE* ε4 carriers (Figure 1). The primary GNPC analysis compared older cognitively unimpaired ε4 carriers (“protected” ε4 carriers) with ε4 carriers affected by AD. To contextualize ε4‐specific findings within broader AD‐related proteomic biology, we also performed population‐wide plasma proteomic analyses across all *APOE* genotypes, modeling diagnostic categories together with *APOE* ε2 and ε4 dosage and *APOE* ε4‐by‐diagnosis interaction terms. Candidate proteins from the protected ε4 analysis were subsequently integrated with external human genetic evidence from gene‐based loss‐of‐function burden testing and MR.

### 2.2 GNPC plasma proteomic cohorts and preprocessing

We used harmonized plasma proteomic data from multiple contributing GNPC cohorts. Analyses were restricted to EDTA plasma study samples. Protein measurements generated on SomaLogic SomaScan platforms were assembled into a unified plasma proteomic matrix, with removal of duplicate analyte columns and recoding −1 to missing. Based on the number of available analytes, samples were classified according to the SomaScan panel version (version 3, 4, or 4.1).

### 2.3 Clinical variable harmonization and diagnostic definitions

To improve comparability across contributing GNPC cohorts, we harmonized measures of clinical severity using Clinical Dementia Rating (CDR) whenever available. For participants without a recorded CDR value, we derived an approximate CDR category from available cognitive screening data using prespecified mappings for the Mini‐Mental State Examination (MMSE) and Montreal Cognitive Assessment (MoCA). MMSE scores of 27 to 30, 21 to 26, 11 to 20, and 0 to 10 were mapped to CDR 0, 0.5, 1, and 2, respectively; the corresponding MoCA ranges were 26 to 30, 18 to 25, 11 to 17, and 0 to 10. Values coded as −1 were treated as missing.

To harmonize diagnoses across contributing cohorts, we used a rule‐based approach centered on clinical severity while retaining a distinct category for non‐AD neurodegenerative disorders. Participants with Parkinson's disease, frontotemporal dementia, or amyotrophic lateral sclerosis were classified as non‐AD neurodegenerative disease. For all other participants, final diagnostic assignment was based primarily on CDR: CDR = 0 defined cognitively normal controls, CDR = 0.5 defined mild cognitive impairment/subjective cognitive impairment (MCI/SCI), and CDR > 0.5 defined AD. If CDR was unavailable, we used the original cohort diagnosis variables, assigning AD when an AD diagnosis flag was present, MCI/SCI when an MCI/SCI flag was present, and control only when no disease flag was present and the available clinical information was consistent with normal cognition. This approach reduced between‐cohort diagnostic heterogeneity, prioritized clinically interpretable severity categories, and preserved non‐AD neurodegenerative disorders for secondary analyses.

For transparency, we recorded the source of clinical‐severity information used in the diagnostic harmonization workflow. Participants were classified as having directly measured CDR when a recorded CDR value was available, as having cognitive‐test‐derived CDR available when CDR was missing but MMSE or MoCA could be mapped to an approximate CDR category, and as requiring original‐diagnosis fallback when neither directly measured nor cognitive‐test‐derived CDR information was available. Diagnostic‐source counts are summarized by analytic cohort and final diagnostic category in Table S1.

### 2.4 *APOE* harmonization and analytic cohorts

*APOE* genotype was harmonized across GNPC cohorts and represented as ε2 and ε4 allele dosages for regression analyses. Sex was similarly harmonized for use as a covariate. The primary protected ε4 analysis focused on *APOE* ε3/ε4 and ε4/ε4 carriers. Protected ε4 carriers were defined as cognitively unimpaired participants at ages when dementia risk is expected to be high among ε4 carriers, specifically ε3/ε4 individuals aged 75 years or older and ε4/ε4 individuals aged 65 years or older. Affected ε4 carriers were defined using the same genotypes but included participants classified as having AD. Accordingly, the primary analysis compared clinically protected older versus affected ε4 carriers to identify protein differences associated with preserved clinical status among genetically high‐risk individuals. For secondary population‐wide analyses, we included all participants with available plasma proteomics, complete covariate data, and one of four harmonized diagnostic categories: cognitively normal control, AD, MCI/SCI, or non‐AD neurodegenerative disease. When repeated visits were available, the last available visit was retained for analysis.

### 2.5 Plasma protein quality control, imputation, and principal components

Protein‐level preprocessing was performed using the cleaned GNPC plasma proteomic matrix and was restricted to EDTA plasma samples. Duplicate analyte columns were removed, sentinel values coded as −1 were recorded as missing, and SomaScan panel version was inferred from the number of measured analytes. We generated both interquartile range (IQR)‐ and 5‐SD‐based quality control (QC) workflows. Both methods were previously used by GNPC with the IQR method used,^20^ while the 5‐SD method was used in Imam et al.^12^ Each workflow was followed by sequential 65% and 85% sample/analyte call‐rate filtering, SoftImpute imputation of remaining missing values, and log10(*x*+1) transformation. Principal component (PC) analysis was then performed on the cleaned log‐transformed matrix; PC‐based outliers were removed using robust Mahalanobis distance in PC space, after which PCs were recomputed for use as covariates in downstream models.

The IQR‐ and 5‐SD‐QC workflows produced highly consistent downstream association results, but the 5‐SD workflow was more sensitive to extreme outliers. We therefore used the IQR‐QC, SoftImpute‐completed, and PC‐filtered plasma dataset for all primary analyses, treating it as the more robust and conservative QC strategy. The first three plasma proteomic PCs were included as covariates because they captured the dominant axes of global proteomic variation after QC and reduced residual structure across cohorts and assay panels. These three PCs explained 62.9% of the total variance in the cleaned plasma matrix (PC1, 57.8%; PC2, 2.7%; PC3, 2.3%). PC1 was strongly correlated with sample mean log10 protein abundance (*r* = 0.970), indicating that the included proteomic PCs captured most global abundance variation. The variance explained by the plasma proteomic PCs and their correlations with sample mean log10 protein abundance are reported in Table S2; sensitivity analyses including mean abundance adjustment and genotype‐ and sex‐stratified models for prioritized proteins are reported in Table S3.

### 2.6 GNPC proteomic association analyses

For the primary protected ε4 analysis, we performed protein‐wise linear regression using HC3 robust standard errors, with log10‐transformed plasma protein abundance as the dependent variable. The primary comparison was between protected and affected ε4 carriers, where affected carriers included participants with AD. Models were adjusted for ε4 allele dosage, age at visit, sex, and the first three plasma proteomic PCs. Multiple testing was controlled using the Benjamini–Hochberg false discovery rate (FDR).

For secondary population‐wide analyses, we performed protein‐wise linear regression across all *APOE* genotypes, again using log10‐transformed plasma protein abundance as the outcome. Covariates included ε4 dosage, ε2 dosage, age at visit, sex, and harmonized diagnostic categories for AD, MCI/SCI, and non‐AD neurodegenerative disease, with cognitively normal controls as the reference group. To assess whether proteomic associations differed according to ε4 status, we additionally included ε4‐by‐AD and ε4‐by‐MCI/SCI interaction terms. The first three plasma proteomic PCs were included in all models. These analyses provided the broader diagnosis‐related and ε4‐modified proteomic signals used for subsequent integration with the protected ε4 analysis.

### 2.7 Integration of protected ε4 and population‐wide proteomic signals

To identify the most robust candidate proteins, we integrated the protected ε4 analysis with the broader GNPC population‐wide proteomic analyses. Specifically, we considered three complementary domains of evidence: proteomic signals associated with AD‐spectrum diagnosis, proteomic signals modified by ε4 status, and proteomic signals associated with protection among ε4 carriers. Within each analysis, statistical significance was defined using a FDR threshold of 0.05. We then evaluated concordance across analyses by examining whether proteins showed nominally significant associations in the same direction of effect across these complementary domains. This integration strategy was designed to prioritize proteins supported both by protected ε4‐specific and more general disease‐related proteomic patterns.

### 2.8 Loss‐of‐function burden analyses

To provide orthogonal human genetic support, we incorporated gene‐based, high‐confidence loss‐of‐function burden results from a population‐scale whole‐genome sequencing analysis across UK Biobank, All of Us, and ADSP.^19^ In that study, Alzheimer's disease and dementia cases and controls were defined within each cohort using prespecified cohort‐specific criteria,^19^ rare coding variants were annotated on GRCh38 using Ensembl VEP with LOFTEE^18^ high‐confidence loss‐of‐function classification, and gene‐based burden testing was performed with REGENIE^21^ with adjustment for age, sex, ancestry PCs, relatedness, and *APOE* ε4 and ε2 dosages. In the present manuscript, these results were used only as external gene‐level support for prioritized GNPC proteins rather than as a primary discovery analysis.

### 2.9 MR analyses

To provide orthogonal genetic support for prioritized proteins, we performed two‐sample MR using pQTLs as exposure instruments and AD genome‐wide association study (GWAS) summary statistics^22^ as the outcome. Plasma pQTL instruments were derived from large‐scale proteogenomic resources including deCODE SomaScan and UK Biobank Olink datasets,^16^ whereas CSF pQTL instruments were obtained from a large CSF proteogenomic meta‐analysis.^15^

For each protein, candidate pQTL instruments were selected from variants associated with protein abundance and present in the AD GWAS dataset. In the plasma analyses, instruments were required to meet a significance threshold of *p* < 5 × 10^−^^8^; in the CSF analyses, a more permissive threshold of *p* < 5 × 10^−^^6^ was used because of the smaller sample size of the CSF pQTL studies. To reduce redundancy and limit bias from correlated signals, instruments were restricted to one variant per 1 Mb region.

Exposure and outcome datasets were harmonized by effect allele before MR analysis. We focused primarily on inverse‐variance weighted (IVW) estimates after exclusion of highly pleiotropic instruments, where pleiotropy was operationally defined as instrument variants associated with more than three proteins across the instrument‐selection framework. When multiple instruments were available, MR results were generated using IVW together with sensitivity methods including weighted median and MR‐Egger. When only a single instrument was available, the causal estimate was obtained using the Wald ratio.

For the current study, we prioritized IVW and Wald ratio results derived from non‐pleiotropic instruments. These MR findings were then integrated with the GNPC proteomic and loss‐of‐function burden results to help prioritize proteins with convergent evidence for a potential role in delaying AD onset.

## 3 RESULTS

### 3.1 Study population and analytic cohorts

After preprocessing and retention of participants present in the final IQR‐based analytic datasets, the population‐wide analysis included 9734 individuals, comprising 4659 cognitively unimpaired participants, 1968 with MCI/SCI, 2251 with AD, and 856 with non‐AD neurodegenerative disease (Table S4). The protected ε4 analysis included 456 protected ε4 carriers and 1096 affected ε4 carriers with AD (Table 1). Across the broader GNPC sample, ε3/ε3 was the most common genotype, whereas ε4/ε4 carriers were enriched among AD cases relative to cognitively unimpaired participants. In the protected ε4 analysis, unaffected participants were older than affected ε4 carriers by design, while the sex distribution was similar between groups. We also summarized the source of diagnostic information used in the harmonization workflow. In the population‐wide IQR analytic set, 5904 of 9734 participants (60.6%) had directly measured CDR available, 2945 (30.3%) had cognitive‐test‐derived CDR available from MMSE or MoCA, and 885 (9.1%) required original cohort diagnosis fallback. In the protected ε4 analytic set, the corresponding counts were 1165 of 1552 (75.1%), 350 (22.6%), and 37 (2.4%), respectively (Table S1).

**TABLE 1:** Demographics of protected ε4 analysis.

	Protected ε4			Affected ε4		
*APOE* genotype	Carriers, *N*	Female, *N* (%)	Age, mean (SD)	Carriers, *N*	Female, *N* (%)	Age, mean (SD)
All	456	271 (59.4%)	79.7 (5.3)	1096	649 (59.2%)	74.7 (11.5)
ε3/ε4	392 (86.0%)	233 (59.4%)	80.6 (4.5)	888 (81.0%)	527 (59.3%)	75.4 (11.6)
ε4/ε4	64 (14.0%)	38 (59.4%)	73.9 (6.2)	208 (19.0%)	122 (58.7%)	71.5 (10.4)

### 3.2 Proteome‐wide landscape of protected ε4 analysis

**FIGURE 2:**
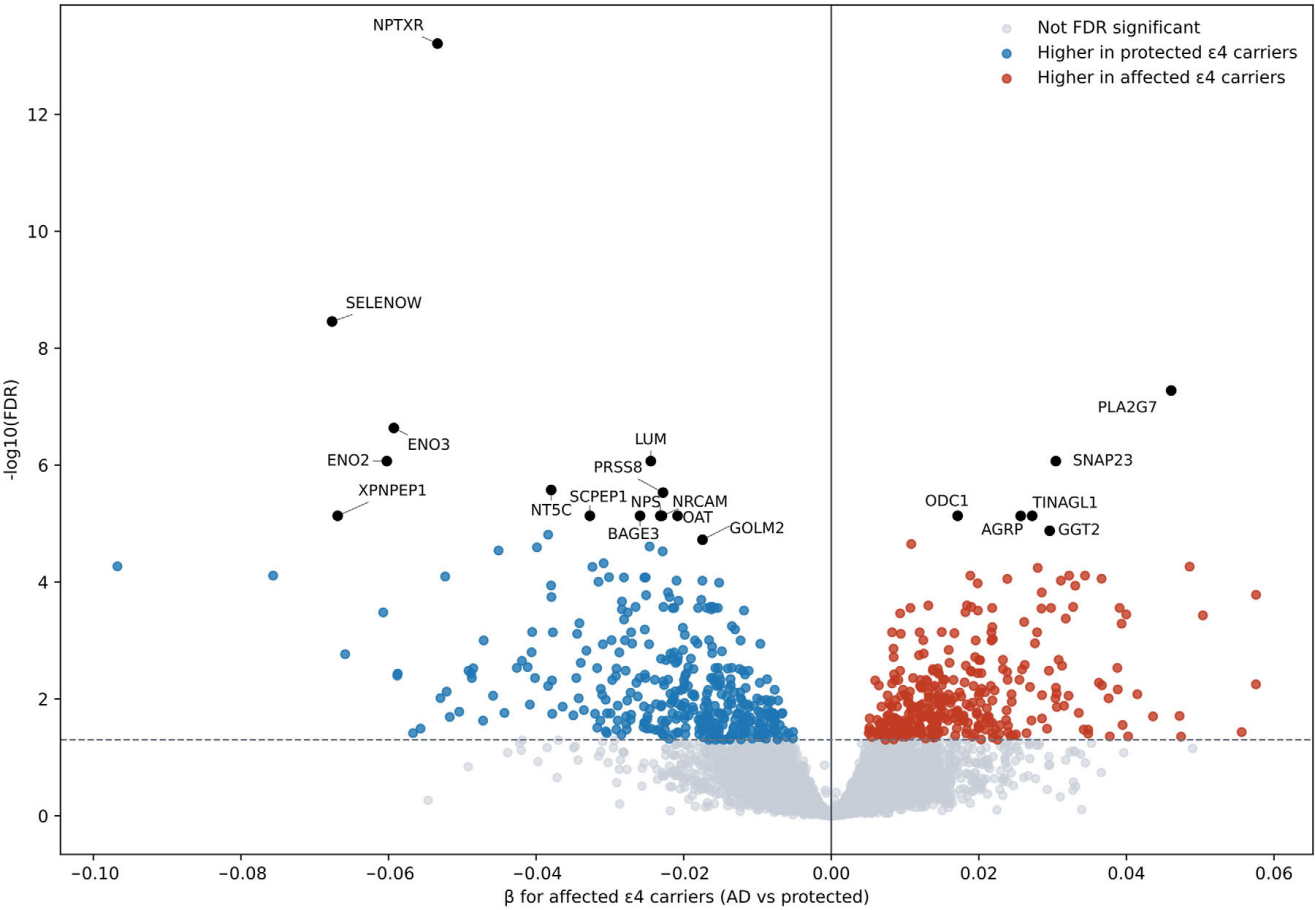
Proteome‐wide landscape of protected ε4. Volcano plot of primary protected ε4 analysis comparing protected APOE ε4 carriers with APOE ε4 carriers with Alzheimer's disease. The *x*‐axis shows the model effect estimate, and the *y*‐axis shows −log10(*p*). Negative *β* values indicate proteins relatively higher in protected ε4 carriers, whereas positive *β* values indicate proteins relatively higher in affected ε4 carriers. The horizontal dashed line denotes the false discovery rate threshold, and selected prioritized proteins are annotated.

The protected ε4 analysis identified a broad plasma proteomic signature associated with preserved cognition among older *APOE* ε4 carriers (Figure 2; Table S5). In total, 721 of 7588 assayed protein measures were associated with protected ε4 at FDR < 0.05, including 386 proteins that were relatively higher in protected ε4 carriers and 335 that were relatively higher in affected ε4 carriers (Table S5). Among the strongest signals enriched in protected ε4 carriers were NPTXR, SELENOW, ENO3, ENO2, LUM, NT5C, and NRCAM, whereas PLA2G7, SNAP23, TINAGL1, ODC1, AGRP, CD81, and APOB were among the strongest signals enriched in affected carriers.

Taken together, these results suggest that protected ε4 is associated with widespread plasma protein differences rather than a limited set of isolated analytes. Broadly, proteins relatively higher in protected ε4 carriers included neuronal, synaptic, and structural candidates, whereas proteins relatively higher in affected ε4 carriers more often reflected inflammatory, lipid‐related, and injury‐associated biology.

### 3.3 Integration with population‐wide GNPC analyses

**FIGURE 3:**
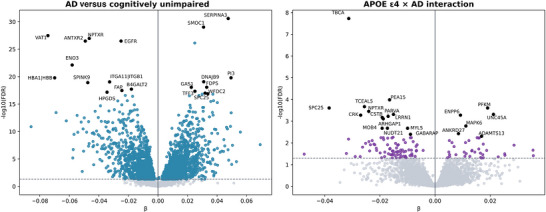
Population‐wide diagnosis and APOE ε4 interaction analyses. Left: volcano plot of population‐wide AD versus cognitively unimpaired comparison across the full GNPC sample. Right: volcano plot of APOE ε4‐by‐AD interaction analysis. The *x*‐axis shows the model effect estimate, and the *y*‐axis shows −log10(*p*). Negative *β* values indicate lower protein levels in AD or in APOE ε4‐by‐AD interaction term, and positive *β* values indicate higher levels. Selected prioritized proteins are annotated. AD, Alzheimer's disease.

In the population‐wide analysis, plasma proteomic differences associated with each of three clinical diagnoses versus controls were widespread (Figure 3; Tables S6 and S7). AD was associated with 3245 protein measures at FDR < 0.05, MCI/SCI with 3153, and non‐AD neurodegenerative disease with 3857. In contrast, *APOE* ε4‐by‐diagnosis interaction effects were much more selective, with 130 significant protein measures for ε4‐by‐AD and only 13 for ε4‐by‐MCI/SCI (Tables S6 and S7). These findings indicate that broad diagnosis‐related proteomic differences are common in plasma, whereas *APOE* ε4‐modified disease effects are comparatively restricted, supporting the value of the protected ε4‐first framework for enriching more specific biology.

Among the strongest AD‐associated proteins in the population‐wide models, SERPINA3 and SMOC1 were increased in AD, whereas VAT1, NPTXR, ENO3, and ANTXR2 were decreased. The strongest ε4‐by‐AD interaction signals included TBCA, PEA15, TCEAL5, PFKM, SPC25, and NPTXR. Taken together, the population‐wide analysis recapitulated broad AD‐related plasma proteomic remodeling while also identifying a smaller subset of proteins whose disease associations differed according to *APOE* ε4 status.

### 3.4 Integration of protected ε4 and population‐wide signals

To place the protected ε4 findings in the context of the broader GNPC disease‐associated proteomic landscape, we examined overlap between proteins associated with protected ε4 and those associated with AD or with ε4‐by‐AD interaction effects in the population‐wide analysis. Of the 721 protein measures associated with protected ε4 at FDR < 0.05, 458 were also significant in the population‐wide AD model, and 440 of these showed concordant direction of effect (Table S8). In addition, 49 were significant in the ε4‐by‐AD interaction model, and all 49 were directionally concordant with the protected ε4 association. When FDR correction was recalculated within the subset of protected ε4‐associated proteins, 474 remained significant for AD and 169 remained significant for the ε4‐by‐AD interaction, again with predominantly concordant directionality. Together, these findings indicate that a substantial proportion of the protected ε4 signal overlaps with broader AD‐related proteomic change, whereas a smaller subset appears to capture more selective ε4‐modified disease biology (Figure S1).

This integrated analysis also helped distinguish proteins associated with general AD‐related biology from those showing more specific evidence of ε4‐modified disease association. For example, NPTXR, VAT1, ENO3, and SERPINA3 were identified both in the protected ε4 analysis and in the broader AD case–control analysis, consistent with a relationship to general disease‐associated proteomic change. By contrast, DBI, BPNT1, PCDH10, and one LILRA5 aptamer showed evidence of ε4‐by‐AD interaction, suggesting that their disease associations may be modified by ε4 status. These results provided the basis for subsequent prioritization of proteins supported across complementary lines of evidence.

### 3.5 Sensitivity analyses of prioritized proteins

We performed sensitivity analyses to evaluate whether prioritized protected ε4 associations were robust to global abundance adjustment and broadly consistent across APOE ε4 carrier subgroups and sex strata. Adding sample mean log10 protein abundance as an additional covariate preserved the direction of effect for all 21 prioritized aptamers, with negligible changes in effect estimates (Table S3). In genotype‐stratified models, effect directions were concordant with the primary protected ε4 model for all 21 prioritized aptamers among ε3/ε4 carriers and for 17 of 21 aptamers among ε4/ε4 carriers. The few discordant estimates in ε4/ε4 carriers were imprecise and not statistically significant, consistent with limited power in the smaller ε4/ε4 subgroup. In sex‐stratified models, effect directions were concordant for 18 of 21 aptamers in males and 20 of 21 aptamers in females. No affected‐by‐sex interaction survived FDR correction. One affected‐by‐APOE ε4‐dosage interaction, for NRBP1, survived FDR correction, whereas a TREM2 interaction was nominal but did not survive multiple‐testing correction. Overall, these sensitivity analyses indicate that the prioritized signals were not driven solely by global mean protein abundance, one APOE ε4 genotype subgroup, or one sex.

### 3.6 Orthogonal genetic support and candidate prioritization

**FIGURE 4:**
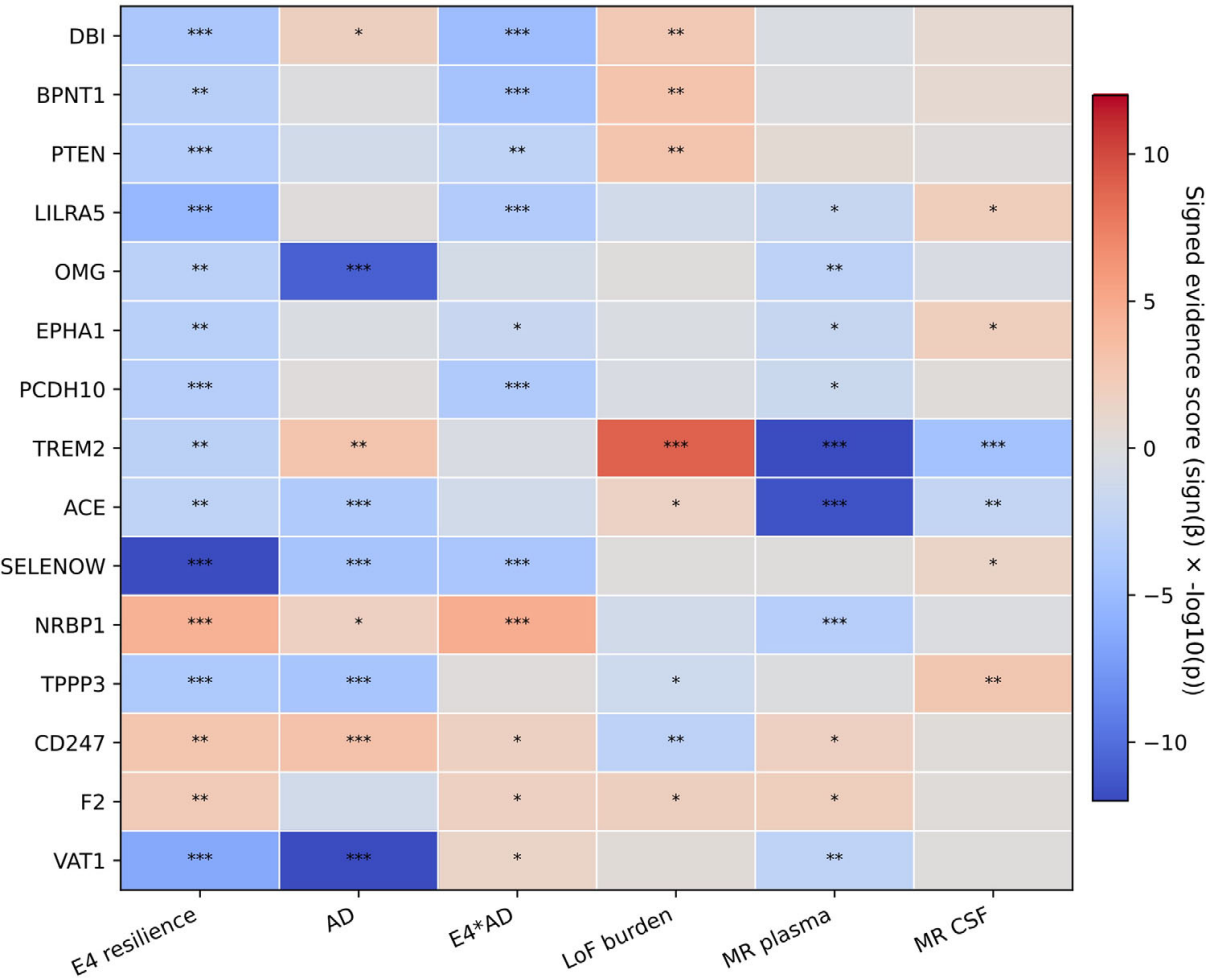
Integrated evidence across proteomic and genetic analyses. Heatmap summarizing direction and strength of evidence for prioritized proteins across protected ε4 analysis, population‐wide AD analysis, APOE ε4‐by‐AD interaction analysis, high‐confidence loss‐of‐function burden testing, and plasma/cerebrospinal fluid Mendelian randomization. Color indicates signed evidence score based on direction of effect and statistical strength. In‐cell significance symbols denote nominal evidence thresholds: *p < 0.05, **p < 0.01, and ***p < 0.001. For the proteomic analyses, false discovery rate significance is reported separately in the corresponding results tables and volcano plots. AD, Alzheimer's disease; MR, Mendelian randomization.

Among proteins associated with protected ε4, several showed convergent support across complementary analytic frameworks, including the population‐wide GNPC models, gene‐based, high‐confidence loss‐of‐function burden testing, and plasma or CSF MR (Figure 4; Table 2). This convergence strengthens prioritization by identifying proteins supported not only by the protected versus affected ε4 comparison, but also by broader disease‐related proteomic patterns and independent human genetic evidence.

**TABLE 2:** Detailed integrated summary of prioritized proteins across proteomic and genetic analyses. For each prioritized protein, the table reports the corresponding effect estimates and *p* values from the protected ε4, AD, and ε4‐by‐AD analyses, high‐confidence loss‐of‐function burden testing results, and the best available plasma and cerebrospinal fluid Mendelian randomization results.

Protein	Protected vs affected ε4	AD	ε4*AD	LoF burden	MR plasma	MR CSF
DBI	−0.020 (*p* = 1.36e‐04)	0.008 (*p =* 0.015)	−0.015 (*p =* 1.52e‐05)	0.712 (*p =* 0.004)	Wald: −0.016 (*p* = 0.478)	IVW: 0.020 (*p* = 0.154)
BPNT1	−0.028 (*p* = 0.001)	−0.000 (*p* = 0.960)	−0.025 (*p* = 5.93e‐05)	0.524 (*p* = 0.001)	IVW: −0.006 (*p* = 0.961)	IVW: 0.016 (*p* = 0.220)
PTEN	−0.021 (*p* = 5.56e‐04)	−0.006 (*p* = 0.103)	−0.012 (*p* = 0.003)	1.235 (*p* = 0.002)	Wald: 0.163 (*p* = 0.253)	IVW: 0.006 (*p* = 0.733)
LILRA5	−0.028 (*p* = 5.20e‐06)	0.002 (*p* = 0.666)	−0.017 (*p* = 3.79e‐04)	−0.258 (*p* = 0.109)	IVW: −0.047 (*p* = 0.013)	IVW: 0.031 (*p* = 0.010)
OMG	−0.031 (*p* = 0.002)	−0.040 (*p* = 1.65e‐11)	−0.010 (*p* = 0.145)	0.104 (*p* = 0.812)	IVW: −0.213 (*p* = 0.003)	IVW: −0.010 (*p* = 0.362)
EPHA1	−0.029 (*p* = 0.002)	−0.004 (*p* = 0.454)	−0.015 (*p* = 0.018)	−0.045 (*p* = 0.509)	IVW: −0.119 (*p* = 0.017)	IVW: 0.019 (*p* = 0.012)
PCDH10	−0.023 (*p* = 6.53e‐04)	0.002 (*p* = 0.719)	−0.018 (*p* = 2.83e‐04)	−0.386 (*p* = 0.407)	IVW: −0.017 (*p* = 0.026)	IVW: 0.011 (*p* = 0.584)
TREM2	−0.025 (*p* = 0.002)	0.018 (*p* = 0.001)	−0.006 (*p* = 0.346)	0.931 (*p* = 1.27e‐09)	IVW: −0.142 (*p* = 3.75e‐23)	IVW: −0.125 (*p* = 4.86e‐05)
ACE	−0.018 (*p* = 0.003)	−0.014 (*p* = 2.57e‐04)	−0.007 (*p* = 0.111)	0.171 (*p* = 0.031)	IVW: −0.145 (*p* = 2.65e‐12)	IVW: −0.080 (*p* = 0.008)
SELENOW	−0.068 (*p* = 9.23e‐13)	−0.024 (*p* = 6.24e‐05)	−0.026 (*p* = 1.13e‐04)	0.045 (*p* = 0.833)		Wald: 0.086 (*p* = 0.044)
NRBP1	0.020 (*p* = 4.29e‐05)	0.007 (*p* = 0.019)	0.015 (*p* = 1.80e‐05)	−1.213 (*p* = 0.077)	IVW: −0.088 (*p* = 7.11e‐04)	IVW: −0.010 (*p* = 0.596)
TPPP3	−0.019 (*p* = 1.95e‐04)	−0.013 (*p* = 9.20e‐05)	0.001 (*p* = 0.736)	−0.515 (*p* = 0.041)	IVW: −0.028 (*p* = 0.745)	IVW: 0.049 (*p* = 0.002)
CD247	0.018 (*p* = 0.002)	0.012 (*p* = 6.73e‐04)	0.009 (*p* = 0.024)	−0.434 (*p* = 0.003)	Wald: 0.251 (*p* = 0.019)	IVW: 0.008 (*p* = 0.539)
F2	0.008 (*p* = 0.005)	−0.003 (*p* = 0.085)	0.005 (*p* = 0.020)	0.550 (*p* = 0.015)	IVW: 0.083 (*p* = 0.011)	IVW: 0.008 (*p* = 0.605)
VAT1	−0.052 (*p* = 3.83e‐07)	−0.074 (*p* = 1.33e‐31)	0.015 (*p* = 0.040)	0.189 (*p* = 0.441)	IVW: −0.097 (*p* = 0.003)	IVW: 0.002 (*p* = 0.887)

Abbreviations: LoF burden, loss‐of‐function burden test; MR, mendelian randomization.

Prioritization was based on convergence across complementary evidence domains rather than on requiring each protein to be significant in every analysis. We therefore interpreted Figure 4 as separating several patterns: proteins with protected ε4 association and ε4‐modified disease effects, proteins with protected ε4 association aligned with broader AD‐related proteomic change, established AD‐relevant positive‐control proteins, and affected‐enriched proteins that may reflect vulnerability, compensatory responses, or context‐dependent disease amplification. This framework allowed concordant findings to strengthen candidate prioritization while allowing biologically interpretable discordant patterns to be retained for cautious discussion.

A first group of proteins combined protected ε4 association with evidence of ε4‐modified disease effects in the population‐wide models. These included DBI, BPNT1, PTEN, LILRA5, EPHA1, PCDH10, NRBP1, CD247, and F2, suggesting that their associations are not limited to general case–control differences but may be particularly relevant to ε4‐associated disease biology. Within this set, DBI, BPNT1, and PTEN also showed supportive high‐confidence loss‐of‐function burden results, whereas LILRA5, EPHA1, PCDH10, NRBP1, CD247, and F2 had additional support from plasma and/or CSF MR. These proteins were not uniform in direction: DBI, BPNT1, PTEN, LILRA5, EPHA1, and PCDH10 were relatively higher in protected ε4 carriers, whereas NRBP1, CD247, and F2 were relatively higher in affected ε4 carriers.

A second group aligned more closely with broader AD‐related proteomic change. OMG, ACE, SELENOW, VAT1, and TPPP3 were all associated with protected ε4 and also showed lower levels in AD in the population‐wide analysis. Within this set, ACE, OMG, VAT1, and TPPP3 had additional support from plasma or CSF MR, and TPPP3 also showed supportive loss‐of‐function burden evidence. In addition, TREM2 provided a strong positive‐control signal, showing association with protected ε4 together with robust support from loss‐of‐function burden testing and both plasma and CSF MR.

Taken together, these findings suggest that the protected ε4‐first framework captures at least two complementary patterns (Figure 4; Figure S2): proteins that may reflect broader AD‐related biology and proteins that may be more informative for ε4‐specific disease modification. Within the latter group, LILRA5 is particularly notable because it combined protected ε4 association, evidence of ε4‐by‐AD interaction in the GNPC models, and supportive MR. Other proteins with a similar ε4‐modified pattern, including DBI, BPNT1, PTEN, EPHA1, and PCDH10, may therefore be especially promising as candidate modifiers of delayed onset among ε4 carriers.

### 3.7 Exploratory pathway enrichment analyses

To provide system‐level context for the protected ε4‐associated proteins, we performed exploratory pathway enrichment analyses using the universe of GNPC‐tested plasma protein genes as background. These analyses were not used as discovery filters but were used to evaluate whether protected ε4‐associated proteins grouped into coherent biological programs. FDR‐significant enrichment was observed primarily among proteins higher in protected ε4 carriers, including terms related to axon development, regulation of axonogenesis, central nervous system development, vascular development, and epithelial–mesenchymal transition/extracellular‐matrix‐associated biology (Table S9). These results are consistent with the broader structural, axonal, vascular, and cell‐adhesion themes emerging from the integrative prioritization framework. Given the exploratory nature of these analyses and the ascertainment properties of plasma proteomic assays, these results should be interpreted as supportive and hypothesis‐generating.

## 4 DISCUSSION

The central contribution of this study is a protected ε4‐first design that focuses on biological mechanisms associated with preserved clinical status in older *APOE* ε4 carriers, rather than on proteins that simply distinguish cases from controls.^12^ This distinction matters in AD plasma proteomics because many case–control signals are likely to reflect downstream neurodegeneration, frailty, vascular comorbidity, or treatment exposure rather than mechanisms that actively delay symptom onset. The broader GNPC literature makes this framing biologically plausible, as carrying *APOE* ε4 is associated with a conserved immune‐related proteomic signature across neurodegenerative diseases, implying that ε4 creates a basal vulnerability state that still requires additional modifiers to shape clinical expression.^12^, ^23^ In this context, the main value of the present results lies in distinguishing proteins that primarily reflect broader AD‐related biology from those that may be more informative for ε4‐specific disease modification.^23^

Within the ε4‐modified tier, LILRA5 is the most immediately compelling hypothesis‐generating candidate. In our data, LILRA5 combined protected ε4 association, ε4‐by‐AD interaction, and supportive MR. The relevance of that pattern is strengthened by a recent large *APOE*‐stratified GWAS, which identified a genome‐wide significant signal at the *LILRA5* locus in the ε4‐positive stratum.^24^ Together, these findings highlight convergence between proteomic ε4‐by‐AD interaction and genotype‐stratified human genetics at this locus. Biologically, *LILRA5* belongs to the leukocyte immunoglobulin‐like receptor cluster on chromosome 19, and recent work shows that *LILRA5* is expressed in naïve monocytes and neutrophils and can trigger reactive oxygen species generation and inflammatory signaling.^25^, ^26^ In a disease context in which *APOE* ε4 already appears to prime chronic immune dysregulation, *LILRA5* is therefore a plausible candidate for ε4‐conditioned innate immune amplification rather than merely a generic inflammatory marker.^23^

*EPHA1* belongs close to *LILRA5* in this interpretive tier, although the underlying biology is different. *EPHA1* is an established AD susceptibility locus from large GWAS, and functional follow‐up of the AD‐associated P460L variant suggests altered receptor activity with consequences for endothelial behavior and blood–brain barrier (BBB) function.^27^ That is relevant here because BBB dysfunction is increasingly recognized as part of AD pathophysiology, and a protected ε4‐associated EPHA1 signal could plausibly index neurovascular protection rather than only amyloid‐ or tau‐centered biology.^28^ *PTEN* is also compelling because its biology sits at the intersection of synaptic signaling, neuronal energetics, and amyloid‐related synaptic toxicity.^29^ Experimental work has shown that Aβ can recruit PTEN to postsynaptic compartments and drive synaptic depression, while human tissue data suggest that aberrant synaptic PTEN increases with symptomatic AD progression.^29^ In the present setting, *PTEN* therefore fits a model in which preserved signaling homeostasis and resistance to synaptic depression may help postpone threshold crossing in ε4 carriers.

PCDH10 adds a complementary synaptic and circuit‐maintenance dimension. *PCDH10* is a protocadherin involved in excitatory synapse development and circuit refinement,^30^ and its association here is consistent with the possibility that the protected ε4 phenotype may involve the preservation of synaptic architecture in addition to the modulation of inflammatory pathways. DBI and BPNT1 are less established in AD, but both are mechanistically plausible and therefore remain biologically relevant candidates in the present framework. *DBI* encodes diazepam‐binding inhibitor/acyl‐CoA‐binding protein, a neuropeptide with links to GABAergic signaling and lipid metabolism, and older clinical work reported altered CSF DBI levels in AD and other dementias. BPNT1 is a lithium‐sensitive 3′‐phosphoadenosine 5′‐phosphate phosphatase with roles in sulfation‐related nucleotide metabolism.^31^ Experimental work has linked BPNT1 inhibition to neuronal dysfunction and lithium‐responsive biology.^32^ *DBI* and *BPNT1* are not established AD susceptibility genes, but their known biology supports their candidacy as markers of metabolic‐stress and neuromodulatory pathways that may be underappreciated in current AD target prioritization.

A second set of proteins appears to align more closely with broader AD‐related plasma remodeling, but several of them remain highly informative. OMG, reduced here in AD versus controls, is particularly important because a new multi‐cohort study now supports it as a brain‐specific proteomic determinant of neurodegenerative resiliency.^33^ In that study, lower plasma OMG was associated with cortical amyloid deposition, compromised brain structure, dementia, and future dementia risk, and genetic analyses supported OMG as causally protective across several neurodegenerative outcomes.^33^ This independent evidence strengthens the interpretation of the present OMG finding and supports its relevance to protected ε4 resilience or resistance‐related biology. SELENOW is also noteworthy because recent experimental work showed that selenoprotein W modulated tau homeostasis and synaptic maintenance in an AD mouse model.^34^ VAT1 is less AD‐specific, but it is a neuronal vesicle protein with oxidoreductase activity and links to vesicular transport, mitochondrial fusion, and phospholipid biology,^35^ making lower VAT1 in AD and relative preservation in protected ε4 carriers biologically plausible. TPPP3 remains a limited‐evidence but intriguing candidate, as it belongs to a tubulin polymerization‐promoting family and has been linked to axon regeneration^36^ and microtubule biology,^37^ which is relevant to neurodegeneration, although evidence directly linking TPP3 to AD remains sparse. . Together, OMG, SELENOW, VAT1, and TPPP3 support the possibility that protected ε4 resilience or resistance is partly mediated by preservation of axonal integrity, synaptic structure, and cytoskeletal stability.^33^, ^34^, ^35^, ^36^

Three proteins merit a more nuanced interpretation because they were relatively higher in affected rather than protected ε4 carriers: NRBP1, CD247, and F2. In our data, all three also showed interaction and/or orthogonal genetic support, arguing against a simplistic “lower in affected equals risk‐increasing” model. NRBP1 is mechanistically relevant because it regulates the degradation of BRI2 and BRI3, physiological inhibitors of APP processing, and Aβ oligomerization, and NRBP1 depletion reduces Aβ production in neuronal cells.^38^ CD247 is the T‐cell receptor ζ‐chain, and although it is not an established AD risk gene, its appearance fits a growing literature implicating adaptive immune^39^ and CD8+ T‐cell alterations in AD progression.^40^ F2 encodes prothrombin, and thrombin is increasingly viewed as a neurovascular and inflammatory mediator in AD that can contribute to BBB dysfunction, microglial activation, and neurotoxicity.^41^, ^42^, ^43^ These genes may therefore prove most useful as markers of ε4‐conditioned vulnerability pathways, compensatory responses, or context‐dependent amplifiers rather than straightforward protective mediators.

TREM2 and ACE occupy a different interpretive category from the less established candidates. In particular, *TREM2* is already strongly anchored in AD pathobiology: Rare coding variants in *TREM2* were among the earliest non‐*APOE* genetic associations to implicate microglial lipid handling and phagocytic function in AD.^44^ Subsequent CSF proteogenomic studies reinforced the relevance of *TREM2*‐related biology to disease mechanisms.^45^ ACE plays a similar positive‐control role; it is a late‐onset AD GWAS locus^46^ and can convert Aβ42 to Aβ40,^47^, ^48^ with *ACE* inhibition enhancing amyloid deposition in experimental systems. The re‐emergence of TREM2 and ACE is therefore reassuring, because it shows that the protected ε4‐first framework recovers established AD‐relevant biology.

Several limitations should be considered. First, a fully independent APOE ε4‐enriched plasma proteomic cohort with comparable clinical definitions and proteomic coverage was not available for replication in this study. The sensitivity analyses by genotype, sex, and mean abundance support the internal robustness of prioritized signals, but independent replication remains essential. Second, amyloid and tau biomarkers were not uniformly available across the contributing GNPC cohorts. We therefore use the term “protected” to describe cognitively unimpaired older ε4 carriers, rather than classifying individuals definitively as resistant or resilient. Future studies integrating amyloid, tau, neurodegeneration, and longitudinal conversion data will be needed to determine whether the proteins prioritized here are associated with lower pathological burden, greater tolerance of pathology, delayed clinical conversion, or some combination of these mechanisms.

From a translational perspective, the most promising proteins are not necessarily those with the largest effect sizes, but those with the clearest convergence across protected ε4 association, ε4‐modified disease association, and human genetic support. On current evidence, LILRA5 stands out as the clearest ε4‐specific hypothesis‐generating candidate, with EPHA1, PTEN, DBI, BPNT1, and PCDH10 forming a second tier of plausible modifiers, while OMG, SELENOW, VAT1, and TPPP3 appear especially informative for a broader axonal and structural‐resilience axis. The next steps should be replication in independent ε4‐enriched cohorts, longitudinal validation that these proteins track delayed conversion rather than survivor bias and integration with amyloid and tau biomarkers to distinguish resistance‐like from resilience‐like mechanisms of protection. More broadly, these data support a meaningful shift toward prevention‐focused AD target discovery.

## CONFLICT OF INTEREST STATEMENT

The authors declare that they have no known competing financial interests or personal relationships that could have appeared to influence the work reported in this manuscript. Author disclosures are available in the Supporting Information.

## CONSENT STATEMENT

Each contributing cohort obtained approval from its local Institutional Review Board (IRB) or Ethics Committee, and all participants gave written informed consent. Human studies were conducted in accordance with the Declaration of Helsinki. The current study protocol was granted an exemption by the Stanford University IRB because the analyses were carried out on deidentified data; therefore, additional informed consent was not required.

## Supporting information




Supporting Information

Supporting Information

Supporting Information
