# Supplementary material for: Proteomic signatures of protected APOE ε4 carriers reveal causal pathways associated with delayed Alzheimer's disease onset
Source: Alzheimers Dement. 2026 Aug 13;22(8):e71688. doi: 10.1002/alz.71688 (PMC13470879; doi:10.1002/alz.71688)
Supplement: Supplementary file 2 [file ALZ-22-e71688-s003.docx]

### **Supplementary Materials**

**Proteomic Signatures of Protected *APOE*-ε4 Carriers Reveal Causal Pathways Associated with Delayed Alzheimer’s Disease Onset**

**Yann Le Guen, PhD^1^, Junyoung Park^2^, Andrés Peña-Tauber^2^, Michael D. Greicius^2^**

1.	Quantitative Sciences Unit, Division of Computational Medicine, Department of Medicine Stanford University, Stanford, CA

2.	Department of Neurology and Neurological Sciences, Stanford University, Stanford, CA

**Corresponding author:**

Yann Le Guen

Quantitative Sciences Unit

3180 Porter Drive

Palo Alto, CA 94304

yleguen@stanford.edu

### **Figure S1. Overlap of *APOE*-ε4 protected signals with population-wide AD and *APOE* ε4-by-AD signals.** Summary of the overlap between proteins associated with *APOE*-ε4 protected and proteins associated with broader AD-related proteomic change or *APOE* ε4-modified disease effects in the population-wide analysis. This figure illustrates the extent to which protected-associated proteins map onto general AD biology versus more selective ε4-modified signals.

**Figure S2. Integrated view of *APOE*-ε4 protected associated proteins across case–control, interaction, and genetic analyses.** Six volcano plots summarize evidence across the primary *APOE*-ε4 protected analysis, the population-wide AD diagnosis analysis, the *APOE* ε4-by-AD interaction analysis, high-confidence loss-of-function burden testing, and plasma and cerebrospinal fluid Mendelian randomization. In the protected panel, all tested proteins are shown, with proteins relatively higher in protected ε4 carriers shown as downward triangles and proteins relatively higher in affected ε4 carriers shown as upward triangles. In the remaining panels, plotting is restricted to proteins that were significant in the *APOE*-ε4 protected analysis at false discovery rate <0.05, and the triangle direction is carried forward from the protected analysis to facilitate visual comparison across analytic frameworks. Colored symbols denote nominally significant associations within each panel, whereas gray symbols denote non-significant associations. The x-axis shows the signed effect estimate and the y-axis shows –log10(q) for proteomic analyses or –log10(p) for burden and Mendelian randomization analyses. Selected top-ranking proteins are annotated in each panel. AD, Alzheimer’s disease; CSF, cerebrospinal fluid; MR, Mendelian randomization.

## **Supplementary tables**

**Table S1. Source of diagnostic information used for clinical harmonization.**
Counts and percentages of participants whose diagnostic classification was supported by directly measured Clinical Dementia Rating (CDR), cognitive-test-derived CDR from MMSE or MoCA, or original cohort diagnosis fallback when CDR information was unavailable. Counts are shown for the population-wide IQR analytic set and the protected-ε4 analytic set, overall and by harmonized diagnostic category.

**Table S2. Variance explained by plasma proteomic principal components and correlation with mean protein abundance.**
Summary of the first plasma proteomic principal components computed after IQR-based quality control, SoftImpute imputation, log10(x+1) transformation, and PC-based outlier filtering. The table reports variance explained by each component and correlations with sample mean log10 protein abundance, supporting the use of proteomic PCs to adjust for global proteomic variation.

**Table S3. Sensitivity analyses for prioritized proteins by APOE genotype, sex, and mean protein abundance adjustment.**
Sensitivity analyses for prioritized protected-ε4-associated proteins. For each prioritized aptamer, the table compares the primary protected-ε4 effect estimate with estimates from models additionally adjusted for sample mean log10 protein abundance, models stratified by APOE ε3/ε4 and ε4/ε4 genotype, models stratified by sex, and interaction models testing affected-by-APOE ε4 dosage and affected-by-sex terms.

**Table S4. Demographic characteristics of the overall GNPC analytic cohort.**
The table summarizes age, sex, and APOE genotype distribution across cognitively unimpaired, MCI/SCI, AD, and non-AD neurodegenerative disease groups in the population-wide GNPC analysis. Values are presented as number, number (percentage), or mean (standard deviation), as appropriate.

**Table S5. FDR-significant proteins in the APOE ε4 protected analysis.**
Full list of protein measures associated with the protected-versus-affected APOE ε4 comparison at false discovery rate <0.05, including effect estimates, standard errors, p values, and adjusted p values.

**Table S6. FDR-significant proteins in the population-wide diagnosis analyses.**
Full list of protein measures associated with AD, MCI/SCI, or non-AD neurodegenerative disease versus cognitively unimpaired status at false discovery rate <0.05 in the population-wide GNPC analysis, including effect estimates, standard errors, p values, and adjusted p values.

**Table S7. FDR-significant proteins in the population-wide APOE ε4-by-diagnosis interaction analyses.**
Full list of protein measures showing significant APOE ε4-by-AD or APOE ε4-by-MCI/SCI interaction effects at false discovery rate <0.05, including effect estimates, standard errors, p values, and adjusted p values.

**Table S8. Overlap of APOE ε4 protected, AD, and APOE ε4-by-AD signals.**
Protein-level overlap between the APOE ε4 protected analysis, the population-wide AD analysis, and the APOE ε4-by-AD interaction analysis, including direction-of-effect concordance across analyses.

**Table S9. Exploratory pathway enrichment analyses of protected-ε4-associated protein sets.**
Exploratory pathway enrichment analyses using the GNPC-tested plasma protein gene universe as background. Enrichment was evaluated for proteins higher in protected ε4 carriers, proteins higher in affected ε4 carriers, all proteins associated with protected-ε4 status at FDR <0.05, and protected-ε4-associated proteins with concordant population-wide AD or APOE ε4-by-AD signals. These analyses were used to provide systems-level context for prioritized signals and were not used as discovery filters.
